# Potential predictors for prognosis and postpartum recovery time of acute fatty liver of pregnancy

**DOI:** 10.1186/s12884-020-03287-y

**Published:** 2020-10-07

**Authors:** Jun Chen, Ze-Bing Huang, Xue-Gong Fan, Xing-Wang Hu, Min Qi, Cheng-Jin Liao, Li-Yuan Long, Yan Huang

**Affiliations:** 1 Department of Infectious Diseases, Xiangya Hospital, Central South University, Kaifu District, Xiangya Road 87, Changsha, 410008 China; 2 Key Laboratory of Viral Hepatitis, Changsha, Hunan China

**Keywords:** Acute fatty liver of pregnancy, Potential predictors, Prognosis, Postpartum recovery time

## Abstract

**Background:**

Acute fatty liver of pregnancy (AFLP) is a potentially lethal condition of pregnant women with a high mortality rate. Potential predictors related to postpartum recovery time and prognostic factors of AFLP are still unclear. This study aimed to evaluate potential predictors for prognosis and postpartum recovery time of AFLP.

**Methods:**

We retrospectively analyzed the clinical data of 76 AFLP patients in our hospital from 2002 to 2017 and investigated potential predictors using univariate analysis and multivariate logistic regression analysis.

**Results:**

Hepatic encephalopathy (HE) was found to be associated with prognosis in AFLP patients (*P* = 0.005, OR = 26.844). The postpartum recovery time analysis showed that AFLP patients with a age < 25 had the shortest recovery time, but no significant difference (*P* = 0.134, OR = 5.952). The postpartum recovery time of patients with liver failure (LF) was significantly prolonged compared to those without LF (*P* = 0.036, OR = 10.052). Cryoprecipitate, and plasma infusion showed no significant effect on prognosis or recovery time. Artificial liver support therapy (ALST) had no effect on prognosis, but it might affect postpartum recovery time with no statistical significance (*P* = 0.128, OR = 5.470).

**Conclusion:**

HE is a potential predictor for prognosis of AFLP. LF is a potential predictor for postpartum recovery time.

## Background

Acute fatty liver of pregnancy (AFLP) is an idiopathic disease occurring mainly in the third trimester of pregnancy and early postpartum period. The 34th gestation week is the critical time for screening AFLP outpatients [1]. Pathologically, AFLP is characterized by hepatocyte fat infiltration, degeneration and necrosis [2]. Clinically, patients often have non-specific clinical manifestations such as fatigue, jaundice, bleeding, and gastrointestinal symptoms. Although the incidence of AFLP is as low as 1/7000 to 1/16,000 pregnancies [3–5], the maternal mortality rate is as high as 7 to 18% [6, 7].

AFLP develop rapidly, causing liver failure (LF) and severe complications which can affect maternal and fetal prognosis and lead to life threatening illness [6, 8]. Currently, different studies showed inconsistent results on the risk factors related to prognosis in patients with AFLP. Meng J reported that total bilirubin (TBIL), prothrombin time (PT), fibrinogen (FIB) and platelet (PLT) were risk factors for the prognosis of patients with AFLP [9]. Zhu TX reported that TBIL, international normalized ratio (INR), serum creatinine (Scr), PLT and hepatic encephalopathy (HE) were related to the prognosis [10]. Moreover, Pan H reported that the time from the visit to termination of pregnancy and from diagnosis to termination of pregnancy were independent risk factors for the prognosis [11].

The most important treatment for AFLP is timely termination of pregnancy with symptomatic and supportive treatment. Liver protection, transfusion of blood components, albumin infusion, infection control, regulation of water and electrolyte balance, and nutritional support are necessary to promote hepatocyte recovery and mitigate complications. Prenatal platelets, total protein and total bilirubin were potential predictors of postpartum recovery time [12]. Artificial liver support therapy (ALST), including blood perfusion, plasma exchange (PE), molecular adsorption recirculation system (MARS) and dual plasma molecular adsorption (DPMAS), has been used in patients with AFLP. However, it is unclear whether it can improve the prognosis or shorten the postpartum recovery time of patients with AFLP.

During clinical diagnosis and treatment, effective measures should be taken to improve prognosis and shorten postpartum recovery time for patients. However, potential predictors for prognosis and postpartum recovery time of AFLP patients are still unclear. Therefore, a full understanding of those potential predictors is vital for the improvement of AFLP patients’ management and the achievement of better clinic outcomes. This study will systematically explore the potential predictors for prognosis and postpartum recovery time in patients with AFLP to provide reference for clinical practice.

## Methods

### Patients

A total of 95 patients with AFLP were admitted to the Department of Infectious Diseases and Obstetrics of our hospital between 2002 and 2017. Patients with known prognosis were admitted into the study of prognosis. Patients who survived were admitted into the study of postpartum recovery time. Meanwhile, patients with other liver injury such as viral hepatitis, tuberculosis, drug-induced liver damage, incomplete clinical data, multiple fetuses (3 or more fetuses) and patients with no abnormalities during pregnancy but diagnosed AFLP after delivery were excluded.

### Diagnostic criteria

The diagnosis of AFLP was based on clinical and laboratory criteria, including symptoms of nausea, vomiting, abdominal pain and polydipsia, characteristic laboratory examination, ultrasound imaging showing fatty liver and liver biopsy. None of the patients underwent liver biopsy, because of the severe conditions, prolonged prothrombin time (PT) and low platelets. All patients exhibited 6 or more of the Swansea criteria [13] (Additional file 1) to confirm the diagnosis of AFLP objectively. Acute kidney injury (AKI) was defined as a Scr > 90umol/L. The diagnosis of disseminated intravascular coagulation (DIC) was based on the DIC score in pregnancy [14]. The diagnostic criteria of LF, HE, and spontaneous bacterial peritonitis (SBP) referred to the latest guidelines or consensus [15–17].

### Collection of clinical data

The data we extracted from medical records were mainly as follows:

1. Demographic data: maternal age, parity, delivery method, number of fetuses, gestational age at diagnosis and time from diagnosis to delivery;
2. Laboratory examination: blood routine, liver function, renal function, blood coagulation routine within 24 h before delivery;
3. Imaging examination: abdominal ultrasound and CT;
4. Main complications: AKI, HE, DIC and SBP;
5. Treatment measures: cryoprecipitate, plasma and ALST (PE, MARS and DPMAS);
6. Prognosis: death or survival;
7. Postpartum recovery time.

### Statistical analysis

Data were analyzed by SPSS v19.0 and GraphPad Prism v7.0 and expressed as mean ± standard deviation (SD) or by frequency and percentage. In the single factor analysis, the t-test for two independent samples was used to compare the measurement data groups that conformed to the normal distribution. The non-parametric test was used to compare the non-normally distributed measurement data groups, and the chi-square test was used to compare the count data groups. Binary logistic regression analysis was used for analysis of potential predictors, and graded data were analyzed by ordered logistic regression. *P* value < 0.05 was considered to be statistically significant.

## Results

### Eligible patients and the general information

**Fig. 1:**
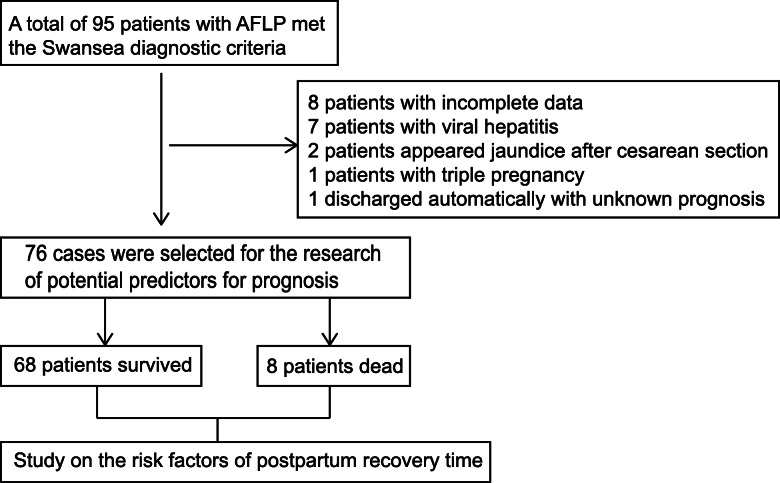
Flow chart of AFLP patients enrolled in the study

Among 95 patients with AFLP, 18 patients were excluded for viral hepatitis, incomplete data, occurring after delivery, and triple pregnancies. One patient was discharged with unknown result. Eight patients died during inpatient. Finally, 76 patients and 68 patients were analyzed for prognosis and recovery time respectively (Fig. 1). B-ultrasound and/or CT showed fatty liver changes in 47 patients. None of the patients had a liver biopsy and liver transplantation. All patients had different degrees of abnormal liver function with elevated total bilirubin and/or transaminase and there was significant separation of bile enzymes (Additional file 2).

### Study on potential predictors for prognosis

The results of univariate analysis showed that TBIL (*P* = 0.014), Scr (*P* = 0.037), and HE (*P* = 0.000) were statistically different, while maternal age, gestational age at diagnosis, time from diagnosis to delivery, number of fetuses, parity, delivery method, white blood cell (WBC), uric acid (UA), PT, cryoprecipitate, ALST, LF, AKI, DIC and SBP were not statistically significant (*P* > 0.05) (Table 1). Then further multivariate logistic regression analysis of TBIL, Scr, and HE showed that HE was potential predictors for prognosis and HE had a significant effect on prognosis for AFLP patients (*P* = 0.005, OR = 26.844) (Table 2).

**Table 1:** Univariate analysis of potential predictors for prognosis

Variable	Survivors (*N* = 68)	Non-svrvivors (*N* = 8)	P
	$\overline{\mathrm{X}}$ ± SD	$\overline{\mathrm{X}}$ ± SD	
Maternal age (Y)	26.84 ± 4.67	26.00 ± 4.75	0.633
Gestational age at diagnosis (W)	34.07 ± 2.97	34.38 ± 1.92	0.912
Time from diagnosis to delivery (d)	12.60 ± 10.45	15.63 ± 12.11	0.540
WBC (×10^9/L)	15.60 ± 7.62	17.88 ± 8.32	0.406
PLT (×10^9/L)	140.32 ± 80.98	86.38 ± 54.64	0.071
TBIL (μmol/L)	146.96 ± 92.70	242.88 ± 169.71	0.014
ALT (U/L)	277.40 ± 281.42	104.75 ± 63.98	0.065
AST (U/L)	306.93 ± 273.48	145.88 ± 67.39	0.080
Scr (μmol/L)	186.85 ± 95.91	254.75 ± 96.93	0.037
UA (μmol/L)	560.53 ± 199.55	380.80 ± 160.68	0.053
PT (S)	23.15 ± 9.32	29.13 ± 11.24	0.138
Cryoprecipitation (U)	16.87 ± 25.05	30.50 ± 40.38	0.578
Plasma infusion (ml)	1147.06 ± 1086.72	1961.25 ± 1492.03	0.089
	n, %	n, %	
Number of fetuses			0.573
1	49, 64.47	5, 6.58	
2	19, 25.0	3, 3.95	
Parity			0.968
primipara	42, 55.26	5, 6.58	
multipara	26, 34.21	3, 3.95	
Delivery method			0.530
Cesarean section	53, 69.74	7, 9.21	
Vaginal delivery	15, 19.74	1, 1.31	
ALST			0.388
YES	18, 23.68	1, 1.31	
NO	50, 65.79	7, 9.21	
LF			0.137
YES	36, 47.37	7, 9.21	
NO	32, 42.16	1, 1.31	
AKI			0.968
YES	63, 82.89	8, 10.53	
NO	5, 6.58	0, 0.00	
HE			0.000
YES	12, 15.79	7, 9.21	
NO	56, 73.68	1, 1.31	
DIC			0.709
YES	33, 43.42	5, 6.58	
NO	35, 46.05	3, 3.95	
SBP			0.808
YES	10, 13.16	2, 2.63	
NO	58, 76.32	6, 7.89	

*ALST* Artificial liver support therapy, *LF* Liver failure, *AKI* Acute kidney injury, *HE* Hepatic encephalopathy, *DIC* Disseminated intravascular coagulation, *SBP* Spontaneous bacterial peritonitis

**Table 2:** Multivariate logistic regression analysis of potential predictors for prognosis

Variable	B	S.E	Wals	P	OR	95% C.I. of EXP (B)	
						Low limit	Upper limit
TBIL	−0.004	0.004	0.904	0.342	0.996	0.988	1.004
Scr	0.001	0.004	0.091	0.763	1.001	0.994	1.008
HE	3.290	1.169	7.920	0.005	26.844	2.715	265.430
Constants	1.150	1.218	0.892	0.345	3.159		

*HE* Hepatic encephalopathy

### Study on potential predictors for postpartum recovery time

The standard of AFLP patients’ recovery: the clinical symptoms relieved obviously, blood routine returned to normal; liver function improved (TBIL and transaminase decreasing to less than twice of the normal upper limit); coagulation function and renal function returned to normal. The period of reaching the recovery criteria after delivery was recorded. Potential factors (including maternal age, gestational age at diagnosis, time from diagnosis to delivery, the number of fetuses, parity, delivery method, ALST, LF, AKI, HE, DIC and SBP) were selected for the analysis of postpartum recovery time. The recovery time was divided into 4 levels during research: fastest recovery (≤7d), faster recovery (8 ~ 14d), slower recovery (15 ~ 28d), slowest recovery (>28d). We used ordinal multi-class logistic regression for analysis (Table 3). The results were as following: (1) Maternal age: Patients aged < 25 years old had the shortest recovery time (*P* = 0.134, OR = 5.985), followed by 25 ~ 29 years old (*P* = 0.457, OR = 2.254). The postpartum recovery time in patients aged 30 ~ 34 years old had no difference compared with those older than 34 years old (*P* = 0.911, OR = 1.140). (2) The gestational age at diagnosis: Patients who developed AFLP at 28th to 37th week of gestation recovered faster than those who developed AFLP after 37th week, but there was no significant difference (*P* = 0.492, OR = 2.012). (3) Patients with single pregnancy, primiparas patients, and vaginal delivery had no significant effect on recovery time compared with twin pregnancy, multipara patients, and cesarean section, respectively (*P* = 0.713, OR = 0.773 VS *P* = 0.440, OR = 0.578 VS *P* = 0.518, OR = 1.634). (4) ALST: ALST could shorten the recovery time, but it was not statistically significant (*P* = 0.128, OR = 5.470). (5) Complications: LF had a significant effect on recovery time. The recovery time in patients without LF had a significant difference compared to those with LF (*P* = 0.036, OR = 10.052). (6) Cryoprecipitate and plasma infusion had almost no effect on postpartum recovery time (*P* = 0.198, OR = 0.982 VS *P* = 0.943, OR = 1.000 respectively). (7) There was no statistical difference in the time from diagnosis to delivery, and it had little effect on postpartum recovery time (*P* = 0.575, OR = 1.018).

**Table 3:** Ordinal logistic regression analysis of potential predictors for postpartum recovery time

	B	SE	P	OR
recovery time > 28 days	−0.502	1.9672	0.798	0.605
recovery time 15 ~ 28 days	3.509	1.9937	0.078	33.400
recovery time 8 ~ 14 days	7.078	2.1617	0.001	1185.730
Maternal age < 25 years	1.789	1.1936	0.134	5.982
Maternal age 25 ~ 29 years	0.813	1.0921	0.457	2.254
Maternal age 30 ~ 34 years	0.131	1.1803	0.911	1.140
Maternal age > 34 years	0^a^	.	.	1
Gestational age 28 ~ 37 week	0.699	1.0177	0.492	2.012
Gestational age > 37 week	0^a^	.	.	1
Single fetal	−0.258	0.7000	0.713	0.773
Twins	0^a^	.	.	1
Primipara	−0.549	0.7114	0.440	0.578
Multipara	0^a^	.	.	1
Cesarean section	0.491	0.7592	0.518	1.634
Vaginal delivery	0^a^	.	.	1
NO LF	2.308	1.1034	0.036	10.052
LF	0^a^	.	.	1
NO AKI	−0.551	1.1839	0.641	0.576
AKI	0^a^	.	.	1
NO HE	0.309	0.9624	0.748	1.362
HE	0^a^	.	.	1
NO DIC	1.110	1.0340	0.283	3.035
DIC	0^a^	.	.	1
NO SBP	−0.987	0.9864	0.317	0.373
SBP	0^a^	.	.	1
ALST	1.699	1.1155	0.128	5.470
NO ALST	0^a^	.	.	1
Cryoprecipitation(U)	−0.018	0.0142	0.198	0.982
Plasma infusion (ml)	2.905E-005	0.0004	0.943	1.000
Time from diagnosis to delivery (d)	0.018	0.0324	0.575	1.018

(^a^This parameter is redundant, so it is set to zero)

*ALST* Artificial liver support therapy, *LF* Liver failure, *AKI* Acute kidney injury, *HE* Hepatic encephalopathy, *DIC* Disseminated intravascular coagulation, *SBP* Spontaneous bacterial peritonitis

## Discussion

AFLP is a rare idiopathic disease with high mortality during pregnancy. AFLP patients usually have non-specificity clinical symptoms. Thus the early diagnosis of AFLP is difficult. Prompt delivery may have the best maternal and fetal outcomes [18–20]. Most AFLP patients recover completely in 1 to 4 weeks after delivery and remain no sequelae if they are terminated of pregnancy timely [21].

Previous studies have shown that TBIL, PT, fibrinogen levels, INR, Plt, Scr and HE are high risk factors for the prognosis of AFLP patients [9, 10]. In this study, multivariate logistic regression analysis finally showed that HE had a significant effect on the prognosis of patients with AFLP. HE is a brain dysfunction secondary to impaired liver function/portal shunt, manifested as neuropsychiatric abnormalities and even being coma [22]. Patients with HE have a poor short-term prognosis with a 1-year survival rate of 42% and a 3-year survival rate of 23% [23]. In our study, 19 patients developed HE, including 7 deaths during hospitalization, with a mortality rate of 36.8%, accounting for 87.5% (7/8) of the deaths. Therefore, in order to improve the prognosis of AFLP patients, measures should be taken to reduce the incidence of HE.

Under the circumstance of liver injury, the decline of synthesis of coagulation factors leads to coagulation dysfunction. Cryoprecipitate and plasma infusion can supplement coagulation factors and correct coagulation dysfunction to some extent. Plasma infusion can supplement blood volume and correct the lack of circulating blood volume caused by postpartum hemorrhage. However, most researchers believe that the cause of AFLP is the non-esterified fatty acid accumulation in pregnancy, which has toxic effects on the liver. After termination of pregnancy, the liver function of most patients can gradually recover. But liver failure would be difficult to reverse if the delivery time was delayed too long causing a large amount of hepatocytes with necrosis. When coming to this condition, it is difficult to improve the prognosis of patients even treating with cryoprecipitate and plasma infusion. Therefore, according to the results of this study, we believe that cryoprecipitate and plasma infusion cannot improve the prognosis of AFLP patients.

ALST has been used in AFLP patients. PE could improve liver function, kidney function and coagulation function for AFLP patients [24, 25]. Moreover, the earlier the PE was performed, the more obvious the effect was, and it was the less likely that ALST would be required again late [26]. However there was a study showing that PE had no significant impact on improving the prognosis and mortality [27]. MARS could improve the clinical symptoms and biochemical parameters of patients with AFLP [28]. MARS can also reduce serum bilirubin levels and improve hemodynamic status, renal function and hepatic encephalopathy in patients with liver failure [29], but it has no effect on the survival of patients with acute liver failure [30]. A study showed the type or number of postpartum ALST sessions and ALST were not related to AFLP patients outcome [31]. In our study, it showed that ALST had no significant effect on the prognosis of patients with AFLP. It may be because of the poor coagulation function, and the use of anticoagulant drugs during ALST may further aggravate coagulopathy and increase the risk of bleeding. AFLP patients with mild liver dysfunction can recover quickly even if there is no ALST. However, for severe patients, the condition can progress rapidly to severe liver failure and it cannot improve liver function even by ALST.

AFLP patients have different recovery time due to individual differences and serious conditions. Patients usually get recovery for 1 to 4 weeks after delivery [21]. This study found that patients younger than 25 years old had the fastest postpartum recovery compared with the other different maternal age, while those older than 29 years had a relatively longer postpartum recovery time. The function of various organs and tissues of the whole body begins to decrease gradually after the age of 35, and the risk of various postpartum diseases is high. In addition, the function of the endocrine to the body and the recovery ability of the reproductive organs will also be weakened after delivery. In general, women’s best reproductive age is between 23 and 30 years old. Patients who developed AFLP on the 28th to 37th weeks recovered faster than those who developed after the 37th weeks. Delivery method and time from diagnosis to delivery had no effects on postpartum recovery time.

AFLP has toxic effects on the liver due to non-esterified fatty acid accumulation, leading to impaired liver function, and can cause complications such as AKI, HE, DIC and SBP. AFLP patients with LF have significantly longer postpartum recovery time than those without LF. The liver acts as a direct target organ for the action of non-esterified fatty acids of toxic metabolites. Our results showed that AKI does not affect the prognosis and postpartum recovery time for patients with AFLP. DIC as serious complications of AFLP can also prolong the postpartum recovery time of patients, but HE and SBP has less effect on postpartum recovery time.

Cryoprecipitate and plasma infusion cannot improve the prognosis of patients with AFLP. ALST can improve the clinical symptoms and organ function of AFLP patients [24, 32]. However, previous study and our data showed that ALST had no significant effect on prognosis [27]. Our study showed that cryoprecipitate and plasma infusion had no significant effect on postpartum recovery time as well. In addition, we found that ALST might shorten the postpartum recovery time, but it was not statistically significant. Bilirubin and other metabolites can be recirculated into the blood after ALST, the symptoms of poisoning in some patients are repeated, and biochemical indicators such as TBIL, ALT, AST, Scr and PT rise, it may take two or more times of ALST. However, due to the high cost of ALST, many patients cannot afford or have the treatment for one time only. So, the effect of ALST on improving prognosis and shortening recovery time is still unclear and further research is needed. Therefore, it needs to consider carefully when performing ALST for AFLP patients.

In conclusion, this study retrospectively analyzed the clinical data of patients with AFLP, and supposed that HE was an important independent risk factor for the prognosis of patients with AFLP and was closely related to the prognosis. In addition, LF had a significant effect on postpartum recovery time for AFLP patients. Interestingly, infusion of plasma or cryoprecipitate and ALST may not improve the prognosis and shorten postpartum recovery time for AFLP patients.

There are still deficiencies in this study. First, this study is a retrospective analysis. The sample is not large enough for this study. Second, the study is limited to one hospital, not a multicenter study. Therefore, the results of this study still need to be further confirmed by more sample data and well-designed prospective studies.

## Conclusions

HE is an independent factor affecting prognosis of AFLP patients. The occurrence of HE carries a poor prognosis. LF is a potential predictor for postpartum recovery time. The earlier of gestational age at diagnosis it is, the shorter time required for postpartum recovery it will be. Cryoprecipitate and plasma infusion have no effect on improving the prognosis and shortening the postpartum recovery time for AFLP patients. ALST has no effect on prognosis, but it may affect postpartum recovery time.

## Supplementary information

**Additional file 1.** Swansea criteria for AFLP.

**Additional file 2.** Biliase separation of AFLP patients. Patients with high TBIL level often have a low transaminase.

## Data Availability

The datasets used and/or analysed during the current study are available from the corresponding author on reasonable request.

## Abbreviations

AFLP: Acute fatty liver of pregnancy
HE: Hepatic encephalopathy
LF: Liver failure
AKI: Acute kidney injury
DIC: Disseminated intravascular coagulation
SBP: Spontaneous bacterial peritonitis
ALST: Artificial liver support therapy
PE: Plasma Exchange
MARS: Molecular Adsorption Recirculation System
DPMAS: Double Plasma Molecular Adsorption
TBIL: Total bilirubin
ALT: Alanine aminotransferase
AST: Aspartate transaminase
PT: Prothrombin time
FIB: Fibrinogen
INR: International Normalized Ratio
WBC: White blood cell
PLT: Platelet
Scr: Serum creatinine
UA: Uric acid

## References

[1] Zhu T, Li Q, Zhang W (2015). Screening time and schedule for outpatients with acute fatty liver of pregnancy. Zhong Nan Da Xue Xue Bao Yi Xue Ban.

[2] Kunelis CT, Peters JL, Edmondson HA (1965). Fatty liver of pregnancy and its relationship to tetracycline therapy. Am J Med.

[3] Liu J, Ghaziani TT, Wolf JL (2017). Acute fatty liver disease of pregnancy: updates in pathogenesis, diagnosis, and management. Am J Gastroenterol.

[4] Wu Z, Huang P, Gong Y (2018). Treating acute fatty liver of pregnancy with artificial liver support therapy: systematic review. Medicine (Baltimore).

[5] Naoum EE, Leffert LR, Chitilian HV (2019). Acute fatty liver of pregnancy: pathophysiology, anesthetic implications, and obstetrical management. Anesthesiology.

[6] Fesenmeier MF, Coppage KH, Lambers DS (2005). Acute fatty liver of pregnancy in 3 tertiary care centers. Am J Obstet Gynecol.

[7] Rajasri AG, Srestha R, Mitchell J (2007). Acute fatty liver of pregnancy (AFLP)--an overview. J Obstet Gynaecol.

[8] Knight M, Nelson-Piercy C, Kurinczuk JJ (2008). A prospective national study of acute fatty liver of pregnancy in the UK. Gut.

[9] Meng J, Wang S, Gu Y (2016). Prenatal predictors in postpartum recovery for acute fatty liver of pregnancy: experiences at a tertiary referral center. Arch Gynecol Obstet.

[10] Zhu TX, Zhang WS, Q L (2016). Analysis of prognostic risk factors in acute fatty liver during pregnancy and establishment of predictive model. Chinese J Clin Med.

[11] Pan H, LJ Z, AB X. (2017). The risk factors on prognosis in acute fatty liver of pregnancy. J Int Obstet Gynecol.

[12] Chen G, Huang K, Ji B (2019). Acute fatty liver of pregnancy in a Chinese tertiary care center: a retrospective study. Arch Gynecol Obstet.

[13] Goel A, Ramakrishna B, Zachariah U (2011). How accurate are the Swansea criteria to diagnose acute fatty liver of pregnancy in predicting hepatic microvesicular steatosis?. Gut.

[14] Erez O, Novack L, Beer-Weisel R (2014). DIC score in pregnant women – a population based modification of the international society on thrombosis and hemostasis score. PLoS One.

[15] Artificial LG, Severe LD, Artificial LG (2019). Guideline for diagnosis and treatment of liver failure. Zhonghua Gan Zang Bing Za Zhi.

[16] Patidar KR, Bajaj JS (2015). Covert and overt hepatic encephalopathy: diagnosis and management. Clin Gastroenterol Hepatol.

[17] European Association For The Study Of The Liver (2010). EASL clinical practice guidelines on the management of ascites, spontaneous bacterial peritonitis, and hepatorenal syndrome in cirrhosis. J Hepatol.

[18] Liu G, Shang X, Yuan B (2016). Acute fatty liver of pregnancy: analysis on the diagnosis and treatment of 15 cases. J Reprod Med.

[19] Mellouli MM, Amara FB, Maghrebi H (2012). Acute fatty liver of pregnancy over a 10-year period at a Tunisian tertiary care center. Int J Gynaecol Obstet.

[20] Cheng N, Xiang T, Wu X (2014). Acute fatty liver of pregnancy: a retrospective study of 32 cases in South China. J Matern Fetal Neonatal Med.

[21] Reyes H, Sandoval L, Wainstein A (1994). Acute fatty liver of pregnancy: a clinical study of 12 episodes in 11 patients. Gut.

[22] Vilstrup H, Amodio P, Bajaj J (2014). Hepatic encephalopathy in chronic liver disease: 2014 practice guideline by the American Association for the Study of Liver Diseases and the European Association for the Study of the liver. Hepatology.

[23] Volk ML, Tocco RS, Bazick J (2012). Hospital readmissions among patients with decompensated cirrhosis. Am J Gastroenterol.

[24] Tang WX, Huang ZY, Chen ZJ (2012). Combined blood purification for treating acute fatty liver of pregnancy complicated by acute kidney injury: a case series. J Artif Organs.

[25] Martin JJ, Briery CM, Rose CH (2008). Postpartum plasma exchange as adjunctive therapy for severe acute fatty liver of pregnancy. J Clin Apher.

[26] Jin F, Cao M, Bai Y (2012). Therapeutic effects of plasma exchange for the treatment of 39 patients with acute fatty liver of pregnancy. Discov Med.

[27] Tang W, Huang Z, Wang Y (2012). Effect of plasma exchange on hepatocyte oxidative stress, mitochondria function, and apoptosis in patients with acute fatty liver of pregnancy. Artif Organs.

[28] Shi XF, Hu Y, WC Z. (2009). Artificial liver system-MARS for treating 14 patients with acute fatty liver of pregnancy. Guanggong Med J.

[29] Yuan S, Zhou Y, Tan D (2011). Impact of heparin on coagulation index during the therapy of molecular adsorbent recirculating system in patients with liver failure. Zhong Nan Da Xue Xue Bao Yi Xue Ban.

[30] Saliba F, Camus C, Durand F (2013). Albumin dialysis with a noncell artificial liver support device in patients with acute liver failure: a randomized, controlled trial. Ann Intern Med.

[31] Wu Z, Huang P, Gong Y (2018). Treating acute fatty liver of pregnancy with artificial liver support therapy. Medicine.

[32] Ding J, Han LP, Lou XP (2015). Effectiveness of combining plasma exchange with plasma perfusion in acute fatty liver of pregnancy: a retrospective analysis. Gynecol Obstet Investig.

