# Supplementary material for: Potential predictors for prognosis and postpartum recovery time of acute fatty liver of pregnancy
Source: BMC Pregnancy Childbirth. 2020 Oct 7;20:601. doi: 10.1186/s12884-020-03287-y (PMC7542749; doi:10.1186/s12884-020-03287-y)
Supplement: Supplementary file 1 — Additional file 1. Swansea criteria for AFLP. [file 12884_2020_3287_MOESM1_ESM.docx]

Additional file 1. Swansea criteria for AFLP

| 1.Vomiting |  |
| --- | --- |
| 2.Abdominal pain |  |
| 3.Polydipsia/polyuria |  |
| 4.Encephalopathy |  |
| 5. Elevated bilirubin | >14µmol/L |
| 6. Elevated transaminases（ALT or AST） | >42IU/L |
| 7. Hypoglycaemia | <4 mmol/L |
| 8. Elevated urate | >340μmol/L |
| 9. Renal impairment: elevated creatinine | >150µmol/L |
| 10. Leucocytosis | >11 x 10^9/L |
| 11. Elevated ammonia | >47µmol/L |
| 12. Coagulopathy：prothrombin time | >14S |
| or activated partial thromboplastin time | >34S |
| 13. Ascites or bright liver on ultrasound scan |  |
| 14. Microvesicular steatosis on liver biopsy |  |
| Six or more of the above features in the absence of another explanation | |
