# Supplementary material for: Potential predictors for prognosis and postpartum recovery time of acute fatty liver of pregnancy
Source: BMC Pregnancy Childbirth. 2020 Oct 7;20:601. doi: 10.1186/s12884-020-03287-y (PMC7542749; doi:10.1186/s12884-020-03287-y)
Supplement: Supplementary file 2 — Additional file 2. Biliase separation of AFLP patients. Patients with high TBIL level often have a low transaminase. [file 12884_2020_3287_MOESM2_ESM.docx]

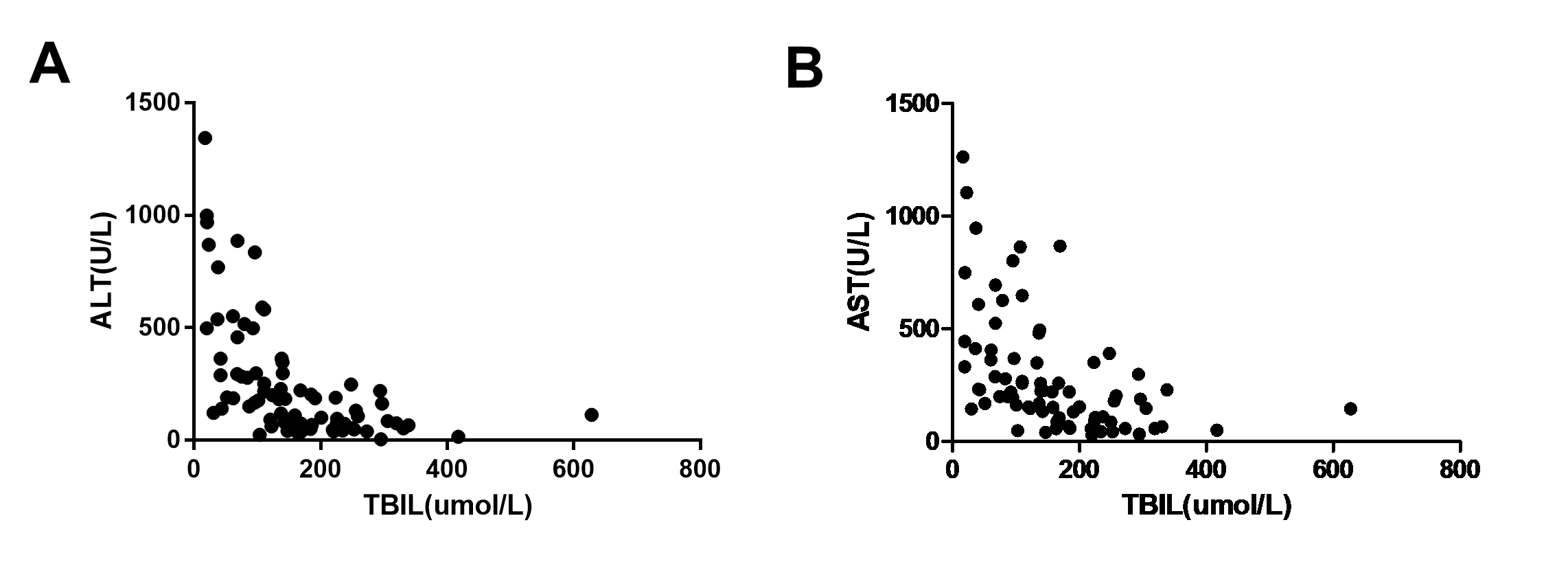


**Additional file 2. Biliase separation of AFLP patients. Patients with high TBIL level often have a low transaminase.** level.
